# Supplementary figures and images for: 72-Hour transport recovery of antimicrobial resistant Neisseria gonorrhoeae isolates using the InTray® GC method
Source: PLoS One. 2022 Jan 21;17(1):e0259668. doi: 10.1371/journal.pone.0259668 (PMC8782362; doi:10.1371/journal.pone.0259668)

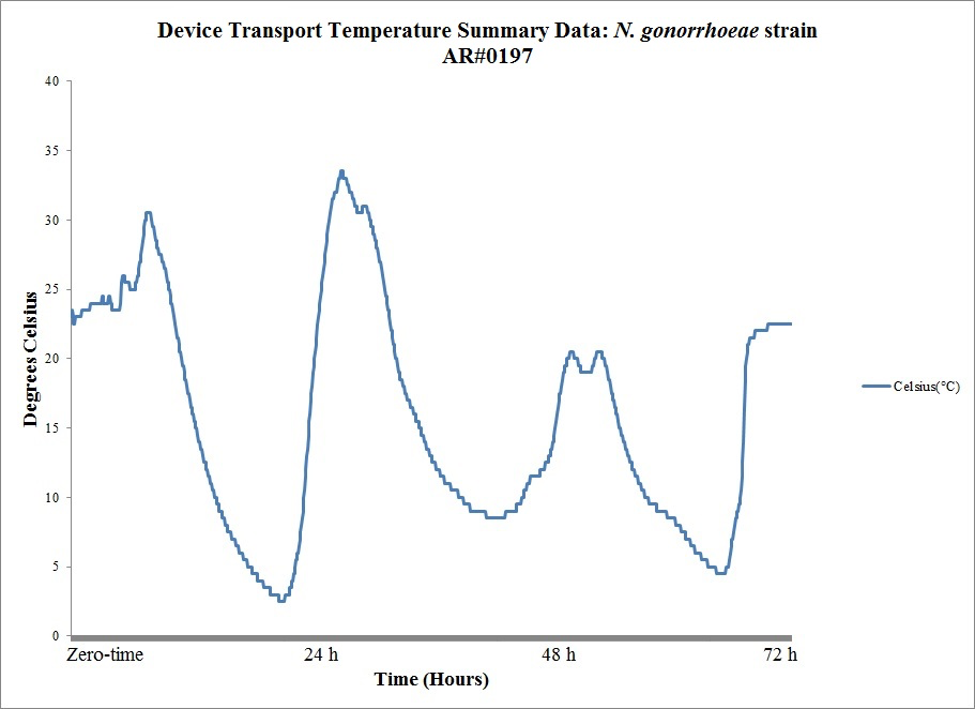

Supplement: S1 Fig — Mean temperature: 15.8°C (Std: 8.3°C), Maximum temperature: 33.5°C, Min temperature: 2.5°C. (TIFF) [file pone.0259668.s001.tiff]

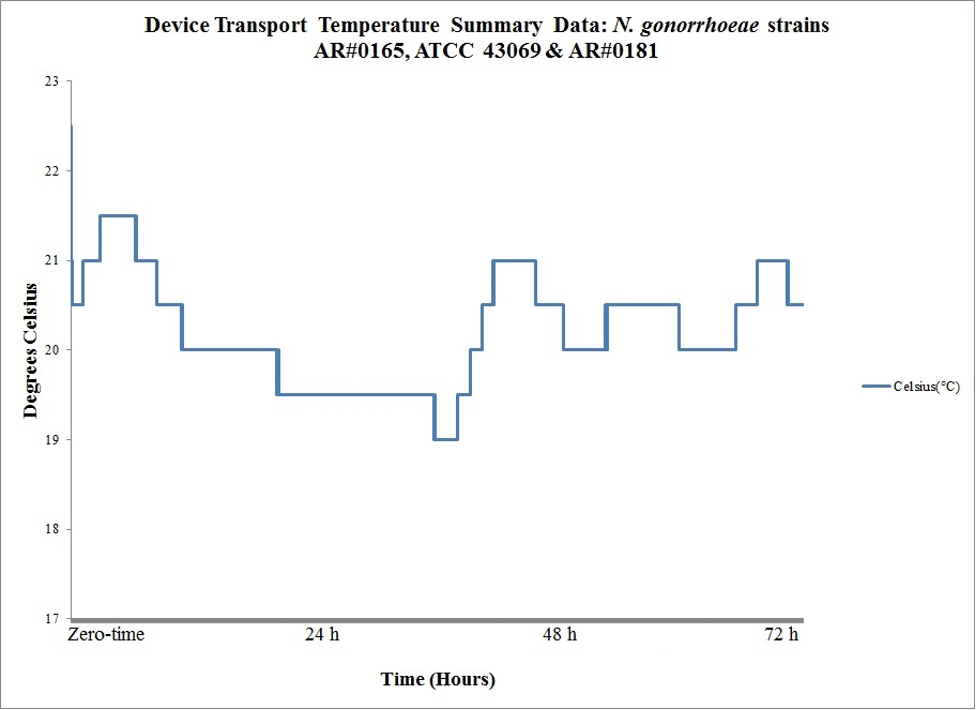

Supplement: S2 Fig — Mean temperature: 20.2°C (Std: 0.6°C), Maximum temperature: 22.5°C, Min temperature: 19°C. (TIFF) [file pone.0259668.s002.tiff]

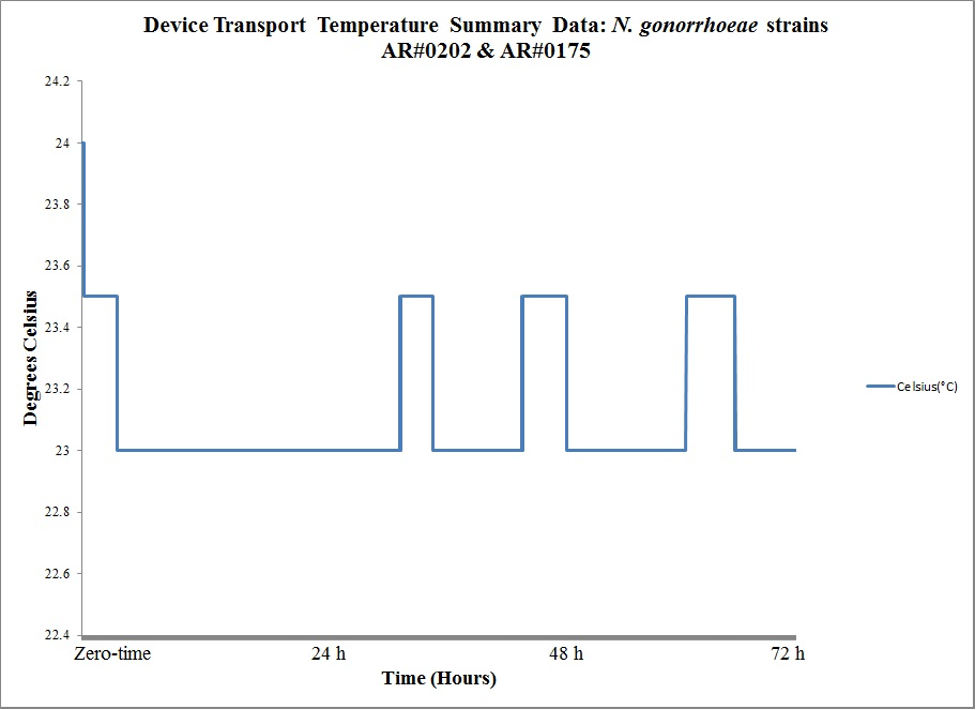

Supplement: S3 Fig — Mean temperature: 23.1°C (Std: 0.2°C), Maximum temperature: 24°C, Min temperature: 23°C. (TIFF) [file pone.0259668.s003.tiff]
