# Supplementary material for: 72-Hour transport recovery of antimicrobial resistant Neisseria gonorrhoeae isolates using the InTray® GC method
Source: PLoS One. 2022 Jan 21;17(1):e0259668. doi: 10.1371/journal.pone.0259668 (PMC8782362; doi:10.1371/journal.pone.0259668)
Supplement: S1 Table — MIC; minimum inhibitory concentration. ATCC 43069 does not have antibiotic susceptibility MIC results. (DOCX) [file pone.0259668.s004.docx]

**SUPPLEMENTAL MATERIAL: Table S1**

**72-Hour Transport Recovery of Antimicrobial Resistant *Neisseria gonorrhoeae* Isolates Using the InTray® GC Method**

Keely S. Paris^1^; Brandon Font^2^; Sanjay R. Mehta^3^ MD; Irvin Huerta^2^, Claire C. Bristow^3^ PhD MPH MSc

*^1^University of California San Diego, La Jolla, CA, USA*

*^2^BioMed Diagnostics Inc., Research & Development, White City, Oregon*

*^3^Division of Infectious Diseases and Global Public Health, Department of Medicine, University of California San Diego, La Jolla, CA, USA*

| **Isolate Number** | **MIC (μg/ml) to Azithromycin** | **MIC (μg/ml) to Cefixime** | **MIC (μg/ml) to Cefpodoxime** | **MIC (μg/ml) to Ceftriaxone** | **MIC (μg/ml) to Ciprofloxacin** | **MIC (μg/ml) to Penicillin** | **MIC (μg/ml) to Tetracyline** | **β -lactamase** |
| --- | --- | --- | --- | --- | --- | --- | --- | --- |
| AR Bank #0165 | 1 | 0.25 | 2 | 0.06 | 8 | 4 | 8 | negative |
| AR Bank #0181 | 256 | 0.06 | 0.125 | 0.03 | 0.015 | 0.5 | 2 | negative |
| AR Bank #0197 | 4 | 0.125 | 0.5 | 0.03 | 16 | 2 | 1 | negative |
| AR Bank #0202 | 16 | 0.015 | 0.015 | 0.008 | 0.015 | 0.25 | 1 | negative |
| AR Bank #0175 | 16 | 0.015 | 0.015 | 0.008 | 0.015 | 0.25 | 1 | negative |

**Table S1.** Minimum inhibitory concentrations (MIC) (in μg/ml) of experimental isolates to azithromycin, cefixime, cefpodoxime, ceftriaxone, ciprofloxacin, penicillin, and tetracycline, as well as the results to a β -lactamase test. MIC; minimum inhibitory concentration. ATCC 43069 does not have antibiotic susceptibility MIC results.
