# Supplementary material for: 72-Hour transport recovery of antimicrobial resistant Neisseria gonorrhoeae isolates using the InTray® GC method
Source: PLoS One. 2022 Jan 21;17(1):e0259668. doi: 10.1371/journal.pone.0259668 (PMC8782362; doi:10.1371/journal.pone.0259668)
Supplement: S2 Table — (DOCX) [file pone.0259668.s005.docx]

**SUPPLEMENTAL MATERIAL: Table S2**

**72-Hour Transport Recovery of Antimicrobial Resistant *Neisseria gonorrhoeae* Isolates Using the InTray® GC Method**

Keely S. Paris^1^; Brandon Font^2^; Sanjay R. Mehta^3^ MD; Irvin Huerta^2^, Claire C. Bristow^3^ PhD MPH MSc

*^1^University of California San Diego, La Jolla, CA, USA*

*^2^BioMed Diagnostics Inc., Research & Development, White City, Oregon*

*^3^Division of Infectious Diseases and Global Public Health, Department of Medicine, University of California San Diego, La Jolla, CA, USA*

| **Transport Device** | ***N. gonorrhoeae*  Seed Inoculum: CFU/mL Concentration (8.11.1)** | **Summary Procedure for Starting Inoculum Volume and Treatment: three replicate devices for zero-time control group (8.11.2)** | **Zero-time Control Group: Summary Plating Procedure for CFU/mL Recovery Quantitation (8.11.3-4)** | **Transport-time Group: CFU/mL Recovery Quantitation (8.11.5)** |
| --- | --- | --- | --- | --- |
| Eswab (flocked nylon fiber swab with 1 mL of liquid Amies medium) | Diluted 0.5 McFarland standard source tube (≈1.5 x 10^8^ CFU/mL) 1:10 in 0.85% saline | Eswab tips were dipped into 100μL of starting inoculum dilution for 10 seconds and placed into 1 mL of liquid Amies medium for 5-15 minutes. Eswabs were vortexed for 10 to 15 seconds and then rotated on the inside of the tube to release maximum quantity of liquid. | 10-fold and 2-fold serial dilutions were prepared from the starting Eswab inoculum from three devices. 90mm chocolate agar Petris were spread-plated with 100μL in duplicate from serial dilutions in order to obtain a countable CFU range (25-250). The average of at least six total plates in the acceptable range (replicates from each of the three devices) was used for CFU/mL calculation^±^. | Same summary plating procedure as described for zero-time, but with three replicate devices after 24 hours of transport. |
| InTray GC (5-6 mL of selective growth medium) | Diluted 0.5 McFarland standard source tube (≈1.5 x 10^8^ CFU/mL) 1:100,000 in 0.85% saline | 20μL of starting inoculum dilution was spread-plated directly onto InTray GC agar and incubated at 35 ± 2°C for 18-to-24 hours. CFUs were counted and recorded at 18-to-24 hours before mixing all colonies into 1.2 mL of 0.85% saline with a 1μL loop. Tubes were vortexed to homogenize. | 10-fold and 2-fold serial dilutions were prepared from the starting InTray GC inoculum from three devices. 90mm chocolate agar Petris were spread-plated with 100μL in duplicate from serial dilutions in order to obtain a countable CFU range (25-250). The average of at least six total plates in the acceptable range (replicates from each of the three devices) was used for CFU/mL calculation^±^. | Same summary plating procedure as described for zero-time, but with three replicate devices after 72 hours of transport. |

**Table S2.**  Inoculation Density and Procedure Adjustments Applied to CLSI M40-A2 section 8.11 Swab Elution Method (Quantitative) for the Eswab and InTray transport GC devices.
